# Measurement of the Effect of Physical Exercise on the Concentration of Individuals with ADHD

**DOI:** 10.1371/journal.pone.0122119

**Published:** 2015-03-24

**Authors:** Alessandro P. Silva, Sueli O. S. Prado, Terigi A. Scardovelli, Silvia R. M. S. Boschi, Luiz C. Campos, Annie F. Frère

**Affiliations:** 1 Technology Research Centre, University of Mogi das Cruzes, Mogi das Cruzes, São Paulo, Brazil; 2 Biomedical Engineering Centre, Pontifícia Universidade Católica de São Paulo, São Paulo, Brazil; University Children's Hospital Tuebingen, GERMANY

## Abstract

Attention Deficit Hyperactivity Disorder (ADHD) mainly affects the academic performance of children and adolescents. In addition to bringing physical and mental health benefits, physical activity has been used to prevent and improve ADHD comorbidities; however, its effectiveness has not been quantified. In this study, the effect of physical activity on children's attention was measured using a computer game. Intense physical activity was promoted by a relay race, which requires a 5-min run without a rest interval. The proposed physical stimulus was performed with 28 volunteers: 14 with ADHD (GE-EF) and 14 without ADHD symptoms (GC-EF). After 5 min of rest, these volunteers accessed the computer game to accomplish the tasks in the shortest time possible. The computer game was also accessed by another 28 volunteers: 14 with ADHD (GE) and 14 without these symptoms (GC). The response time to solve the tasks that require attention was recorded. The results of the four groups were analyzed using D'Agostino statistical tests of normality, Kruskal-Wallis analyses of variance and post-hoc Dunn tests. The groups of volunteers with ADHD who performed exercise (GE-EF) showed improved performance for the tasks that require attention with a difference of 30.52% compared with the volunteers with ADHD who did not perform the exercise (GE). The (GE-EF) group showed similar performance (2.5% difference) with the volunteers in the (GC) group who have no ADHD symptoms and did not exercise. This study shows that intense exercise can improve the attention of children with ADHD and may help their school performance.

## Introduction

Attention Deficit Hyperactivity Disorder (ADHD) is primarily characterized by inattention, restlessness (hyperactivity) and impulsivity. Children and adolescents with ADHD have some impairment in their social and school life throughout development such as poor academic performance, repetition, suspension, difficult relationship with family and peers and low tolerance for frustration [1].

Among the comorbidities, studies relate the body weight [2], cognitive deficit [3, 4] and motor difficulties with ADHD [1]. Children with ADHD presents more risk behavior than those without this disorder [5].

Treatment usually consists of guidelines for parents and teachers with or without drug administration [6, 7, 8]. However, according to recent studies [9], exercise provides beneficial changes such as decreased risk of chronic degenerative diseases, increased bone mineral density and cardiorespiratory benefits. Recently, improvement in cognitive function was also discovered. The hormones that regulate memory (catecholamines) are also involved in the homeostatic regulation of exercise [9, 10]. These surveys showed that the release of catecholamines, vasopressin, ACTH and B-endorphin is stimulated by exercise, which proves the relationship between intense exercise and memory regulation.

Several researchers have sought to determine the influence of external stimuli on attention in children with ADHD. Studies have shown that specific colors [11] and even body vibration [12] are relevant to the performance of people with this disorder.

Therefore, the hypothesis that the attention of ADHD children can be improved using physical activity has been proposed. Therapists have indicated physical activity to prevent several comorbidities [9, 13]; however, there is no quantitative comparison of the obtained benefits.

Thus, this work aims to quantify the effect of physical activity on the attention of children with ADHD.

## Methods

### Ethical approval

Approval was obtained from the Ethics Committee in Research to involve humans at the University of Mogi das Cruzes (CAAE-0151.0.237.000–10, process CEP/UMC-157/2010) as the participants. They were informed about the methodology and confidentiality of their personal information. All procedures were performed after the informed consent was written by the school’s director and the guardians on behalf of the children according to the institutional ethics guidance.

### Research Subjects

Sixty-six volunteers of 10–16 years of age participated in the selection of City College São Simão—Goiás. This age group was chosen because of the ease of performing intense exercise. Among these volunteers, 38 were diagnosed with ADHD.

The ADHD diagnosis was conducted by a psychologist with expertise in clinical psychology, who evaluated the reports of school performance, history of the students, parents’ reports and test results [14, 15].

The choice of the volunteers strictly followed the criteria for inclusion or exclusion in the study, which are described below.

The criteria for inclusion of volunteers with ADHD are as follows:

1. have parental consent to participate in the study;
2. be of an age between 10 and 16 years;
3. be regularly enrolled in an educational institution;
4. have no comorbidities that are not related to ADHD (e.g., OCD, phobias, stress syndromes);
5. not currently have drug treatment;
6. have ADHD diagnosis for more than six months;
7. have a medium level of performance (300 sec ± 5%) in the commercial computer game “Prince of Persia”.

The volunteers were selected by evaluating their computer skills in executing one introductory level of the commercial computer game “Prince of Persia” [16] with similar commands and characteristics to the computer game “Raiders of the Lost Treasure” [11], which was applied to evaluate the attention of the volunteers. Each user could define which commands to use: the mouse and keyboard simultaneously or only the keyboard. From the players who accomplished this test in 300 sec ± 5%, 76 volunteers were recruited, among whom were 38 non-ADHD volunteers (control group) and 38 volunteers with ADHD, free of medications.

Among the 38 volunteers with ADHD, one was dismissed for having other comorbidities, and two left the school after the interviews. Seven were dismissed because of problems in playing the game “Prince of Persia” to avoid repetition from affecting the final results.

Twenty-eight volunteers were selected for the control group: volunteers with notably good grades in all subjects, without reports about behavior problems and a good social life at school and at home.

The criteria for inclusion of volunteers without ADHD are as follows:

1. have parental consent to participate in the study;
2. be between 10 and 16 years;
3. be regularly enrolled in an educational institution;
4. have a report on good behavior and interest in the classroom;
5. not have his/her name included in school reports about behavior problems;
6. have parents' reports about a good social life at school and at home.
7. have a medium level of performance (300 sec ± 5%) in the commercial computer game “Prince of Persia”.

The volunteers were divided into two groups: 28 volunteers in the group with ADHD and 28 without symptoms in the control group. The group of students with ADHD was divided into two groups: GE-EF, who were volunteers with ADHD and performed physical activity, and GE ADHD, who were volunteers with no physical activity. The control group was subdivided into GC-EF, which was the control group with physical activity, and GC, which was the control group with no physical activity, both of which had 14 volunteers.

### Protocol

The following protocol was used to verify whether physical exercise improves the attention of the students with ADHD:

Initially, each volunteer was interviewed while accompanied by his or her parents. The objectives and procedures of the study were explained to the volunteers, and they were asked to sign the ethical terms. Additionally, some identification data of the volunteers were collected to characterize the sample, such as age and sex, and the basal heart rate was measured to calculate the target training zone. A pilot test was conducted to characterize whether the selected physical stimulus was sufficient to achieve the training-zone heart rate. Thus, the volunteers underwent a simplified relay race with pulse measurement 3 and 5 min after the activity. This measurement was performed for all volunteers, even those who were not involved in the physical test.

A month after the basal heart rate record was measured, a relay race was proposed as the intense physical activity. The GE-EF and GC-EF groups attended the proposed physical activity and subsequently performed the computer game “Raiders of the Lost Treasure,” which evaluated the level of attention. The volunteers from groups GE and GC did not participate in the proposed physical activity and only used the computer game “Raiders of the Lost Treasure” to test their attention span. The material in the relay race was inflatable balloons and large plastic bags. The volunteers were lined up at one end of the court, and inflatable balloons were placed opposite to them. Each volunteer had a plastic bag set in a holder at his side. Each volunteer had to run and catch an inflatable balloon, bring and put it in his plastic bag and return to catch another balloon. The winner was the one who gathered the greatest amount of balloons in 5 min.

The volunteers rested for 5 min and played the computer game “Raiders of the Lost Treasure,” which was developed in a previous study [11], to quantify the performance of people with ADHD characteristics. To overcome the challenges, the player had to read and interpret the hints in the plot in addition to focusing on the scenario details. The principal metric evaluation that was performed by the investigators identified the time the volunteer took for the task until completion. According to the authors, this game follows the APA recommendations (American Psychiatric Association) not to discourage people with ADHD by have characteristics such as shorter phase, higher frequency of rewards and exchange of visual stimuli.

**Fig 1:**
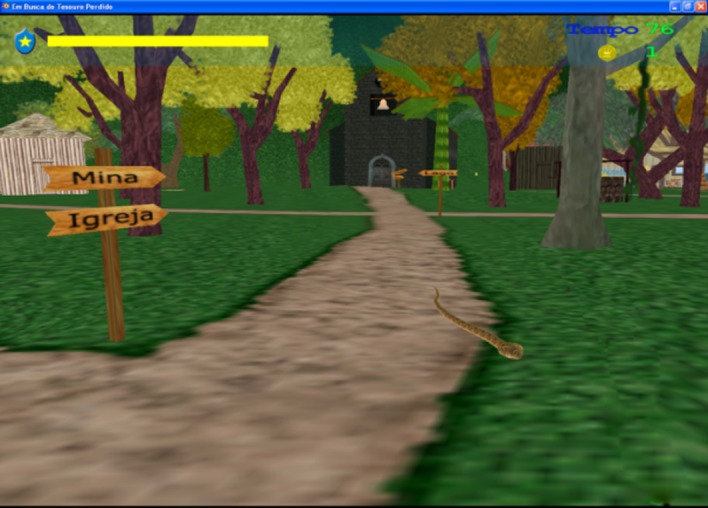
External Environment [6].

**Fig 2:**
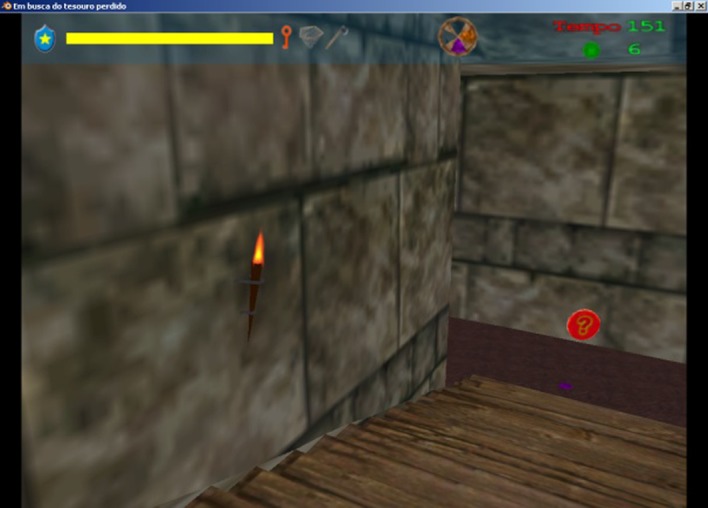
Internal Environment [6].

This game was divided into two phases: the 1st phase was set in an island (external environment) (Fig 1), and the 2nd phase was set in a mine (internal environment) (Fig 2).

**Fig 3:**
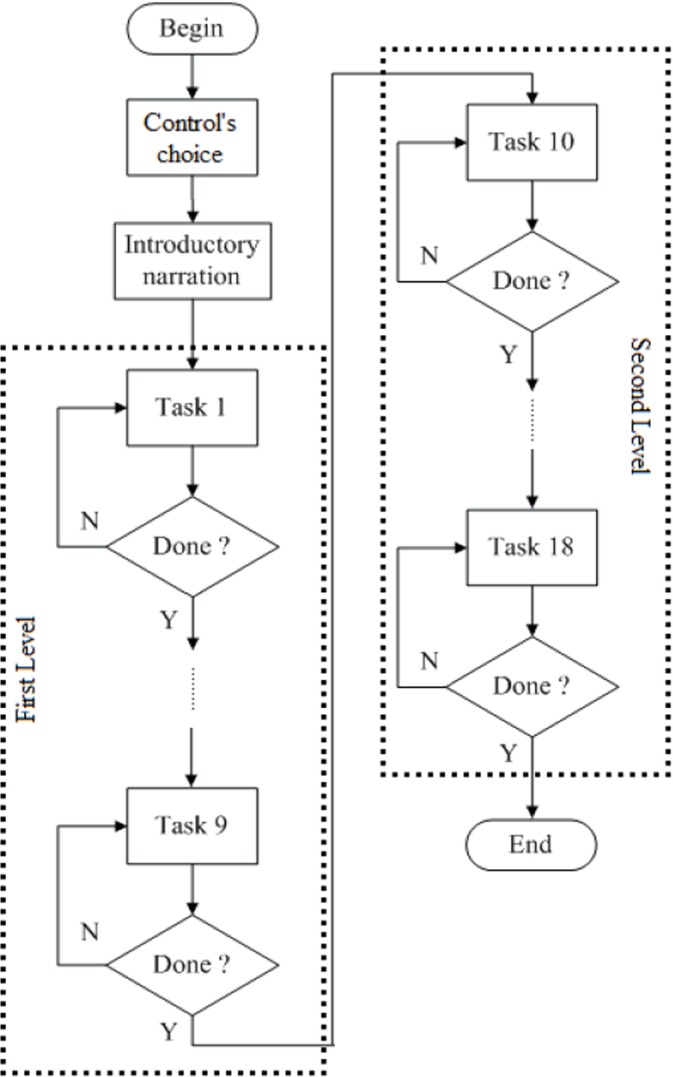
The game’s logical diagram [6].

The game was strategically developed into sequential modules with embedded tasks, which followed a predefined and gradual logical order (Fig 3).

Table 1 presents the description of the game’s modules and their respective tasks.

**Table 1:** Game’s task description.

Level one	Task 1	Find the resident who knows the secrets of Treasure Island;
	Task 2	Find the trunk where the first part of the mandala is located;
	Task 3	Find the key that opens the trunk;
	Task 4	Go back to the trunk and catch the first part of the mandala;
	Task 5	Find the lake where the second part of the mandala is submerged;
	Task 6	Find a fishing net to catch the second part of the mandala;
	Task 7	Return to the lake to catch the second part of the mandala;
	Task 8	Locate the mine where the other parts of the mandala resides;
	Task 9	Find the axe to break the fence that blocks the mine entrance;
Level two:	Task 10	Find the wizard who knows where the third part of the mandala is located;
	Task 11	Find the place indicated by the wizard;
	Task 12	Frighten the owl that guards the third part of the mandala;
	Task 13	Find the wizard, who then requests the player to locate his lost ring in exchange for the fourth part of the mandala;
	Task 14	Find the wizard’s ring;
	Task 15	Find a piece of meat to capture the tiger that guards the wizard’s ring;
	Task 16	Return to the wizard and give him the ring to receive the fourth part of the mandala;
	Task 17	Find the treasure room;
	Task 18	Finally, insert the completed mandala into the hole on the wall close to the treasure room’s door to unlock it.

### Calculating the training target zones

To calculate the aerobic threshold of each volunteer, Karvonen method was used [14, 16]. For both volunteer groups, the maximum aerobic threshold as a function of the maximum heart rate was calculated to define the maximum target zone (Zamax). The minimum target zone (Zamin), which is defined as the ratio between the minimum aerobic threshold and the minimum heart rate, was also calculated. The heart rate in the target zone of training is defined as the heart rate that swings between the minimum target zone and the maximum target zone.

### Statistical analysis

A normality test was applied to evaluate the data sampling distribution. In addition, analyses of variance allowed us to identify differences among the groups (p-value ≤ 0.05). The test post-hoc was used to define which group differs from the remainder of the sample.

The programming module of the Matlab software was used to analyze the complete game data as a function of time, with which the routine had to generate continuity of pre-written results because these information is discretely store.

## Results

The task of the relay race was performed with GE-EF and GC-EF on different days but at the same hour. Each volunteer ran without rest for 5 min and took an average of 15 balloons. After the physical exercise, the volunteers underwent a test match, and the time was recorded. Table 2 presents the sum of spend time for each task of game by all groups.

**Table 2:** Sum of spend time for each task of game by all groups.

Tasks	GE-EF		GC-EF		GE		GC	
Task 1	297,37	± 3%	1226,07	± 6,34%	733,77	± 2,06%	365,70	± 2,41%
Task 2	385,29	± 5,72%	1038,30	± 3,85%	590,12	± 0,65%	435,72	± 2,13%
Task 3	634,89	± 1,12%	1787,97	± 5,37%	1082,36	± 2,36%	1051,19	± 2,52%
Task 4	168,40	± 2,01%	396,00	± 3,52%	287,66	± 1,81%	227,98	± 2,11%
Task 5	255,41	± 1,83%	610,80	± 3,11%	296,53	± 1,67%	300,16	± 1,58%
Task 6	203,19	± 2,66%	727,61	± 3,35%	516,27	± 4,54%	384,57	± 2,04%
Task 7	156,56	± 2,68%	380,84	± 4,54%	220,14	± 1,15%	237,77	± 2,09%
Task 8	296,73	± 3,80%	786,00	± 4,19%	467,67	± 2,49%	422,92	± 2,19%
Task 9	582,03	± 3,50%	1229,39	± 3,13%	622,61	± 1,36%	621,31	± 2,28%
Task 10	307,21	± 1,37%	370,45	± 2,54%	714,74	± 7,18%	199,98	± 0,88%
Task 11	268,96	± 1,97%	243,35	± 2,21%	464,39	± 2,74%	188,28	± 1,42%
Task 12	495,87	± 3,73%	401,99	± 3,28%	527,43	± 2,59%	303,45	± 3,14%
Task 13	309,67	± 1,57%	364,77	± 2,08%	581,53	± 4,31%	235,78	± 3,63%
Task 14	291,44	± 1,50%	489,13	± 3,96%	442,27	± 2,09%	271,25	± 2,10%
Task 15	689,10	± 0,68%	596,34	± 3,33%	844,30	± 1,88%	452,56	± 1,84%
Task 16	197,98	± 1,59%	137,71	± 0,84%	261,66	±2,85%	129,14	±1,56%
Task 17	836,98	± 1,36%	681,01	± 2,29%	762,11	± 2,03%	599,07	± 0,89%
Task 18	990,06	± 0,52%	875,77	± 2,97%	1183,67	± 1,85%	755,93	± 1,23%

Table 3 shows the average time (in seconds) that was taken for each task group in the game.

**Table 3:** Average time (seconds) taken by the participants of the groups in each game’s task.

Tasks	GE-EF	GC-EF	GE	GC
Task 1	21.24	87.58	52.41	26.12
Task 2	27.52	74.16	42.15	31.12
Task 3	45.35	127.71	77.31	75.08
Task 4	12.03	28.29	20.55	16.28
Task 5	18.24	43.63	21.18	21.44
Task 6	14.51	51.97	36.88	27.47
Task 7	11.18	27.2	15.72	16.98
Task 8	21.19	56.14	33.4	30.21
Task 9	41.57	87.81	44.47	44.38
Task 10	21.94	26.46	51.05	14.28
Task 11	19.21	17.38	33.17	13.45
Task 12	35.42	28.71	37.67	21.68
Task 13	22.12	26.05	41.54	16.84
Task 14	20.82	34.94	31.59	19.38
Task 15	49.22	42.6	60.31	32.33
Task 16	14.14	9.84	18.69	9.22
Task 17	59.78	48.64	54.44	42.79
Task 18	70.72	62.56	84.55	53.99

Table 4 shows the respective relative percentages for the participants to complete each task in the function of the GC group.

**Table 4:** Relative percentages for each game’s task by all groups.

Tasks	GE-EF	GC-EF	GE	GC
Task 1	81	335	201	100
Task 2	88	238	135	100
Task 3	60	170	103	100
Task 4	74	174	126	100
Task 5	85	203	99	100
Task 6	53	189	134	100
Task 7	66	160	93	100
Task 8	70	186	111	100
Task 9	94	198	100	100
Task 10	154	185	357	100
Task 11	143	129	247	100
Task 12	163	132	174	100
Task 13	131	155	247	100
Task 14	107	180	163	100
Task 15	152	132	187	100
Task 16	153	107	203	100
Task 17	140	114	127	100
Task 18	131	116	157	100

**Fig 4:**
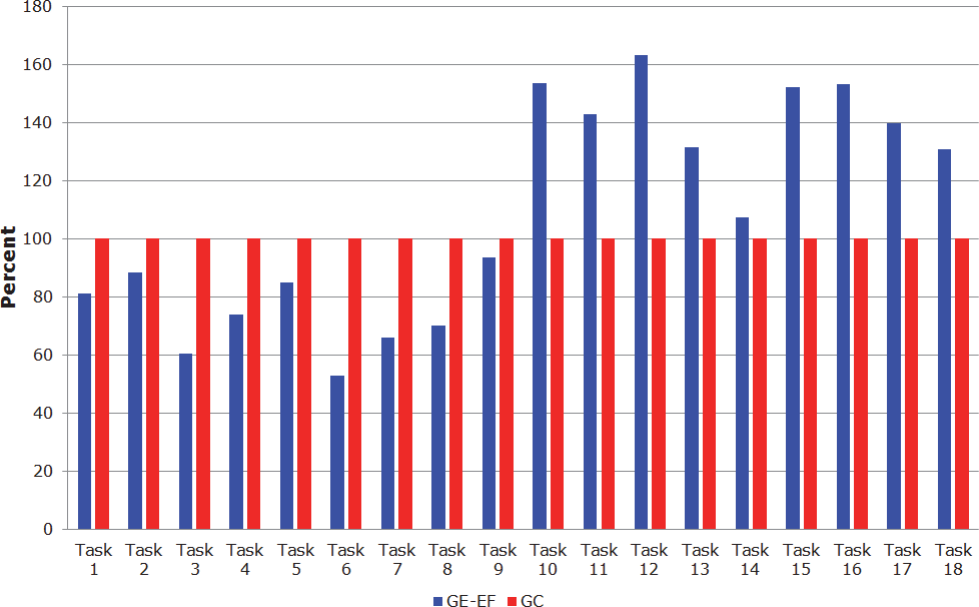
Graph by sum of average time taken by group GE-EF and GC to complete the game.

**Fig 5:**
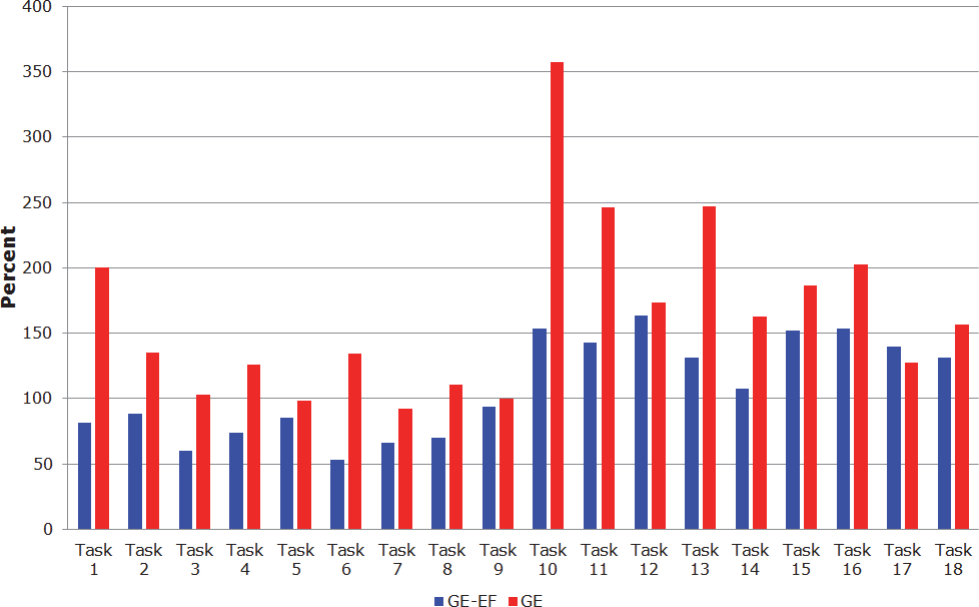
Graph of percentage by group GE-EF and GE to complete the game.

The GE-EF and CG groups showed approximately the identical performance compared to the complete game, with a small standard deviation, which proves the sample homogeneity. Fig 4 shows the percentage differences between GE-EF and GC. Fig 5 shows the percentage difference between GE-EF and GE in the entire game.

Table 5 shows the sum of the average total duration of each group to complete the game.

**Table 5:** Sum of the average time all groups in the full game.

groups	Average Time (s)
GE-EF	526
GE	757
GC-EF	882
GC	513

**Fig 6:**
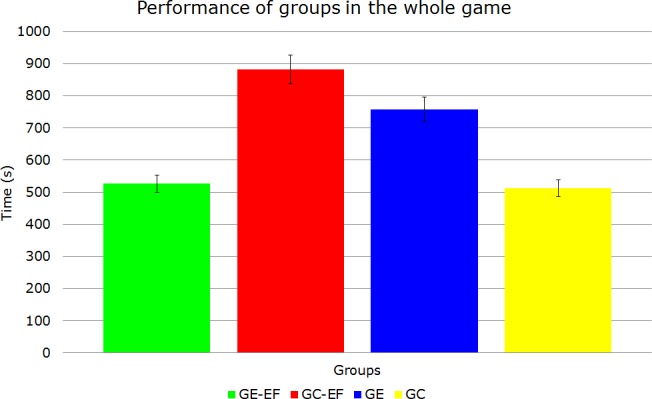
Graph of the average performance of the 4 groups.

Fig 6 shows the sum with the standard deviation of each group to complete the game.

The group with ADHD and that participated in the proposed physical activity (GE-EF) obtained 30.52% better performance than the group with signs of ADHD that did not participate in physical activity (GE) and 40.36% better than the group without characteristic of ADHD that participated in physical activity (GC-EF). The group that more rapidly reached the goal proposed by the assessment game was that group without characteristic of ADHD and not involved in physical activity proposed (GC), ending the testing game 2.5% faster than the GE-EF group.

Regarding the normal distribution analysis, D'Agostino test was applied allowing that the investigators identify that the sampling distribution is non-parametric. In addition, Kruskal-Wallis test was used showing significant differences between groups (H = 32.2207, p ≤ 0.0001). It was followed by the Post-hoc Dunn test, where significant differences between groups were also identified. See Table 6 for details.

**Table 6:** Statistical analysis using the method Dunn.

Comparison using method Dunn	p
GE-EF versus GC-EF	< 0,05
GE-EF versus GE	< 0,05
GE-EF versus GC	ns
GC-EF versus GE	ns
GC-EF versus GC	< 0,05
GE versus GC	< 0,05

**Fig 7:**
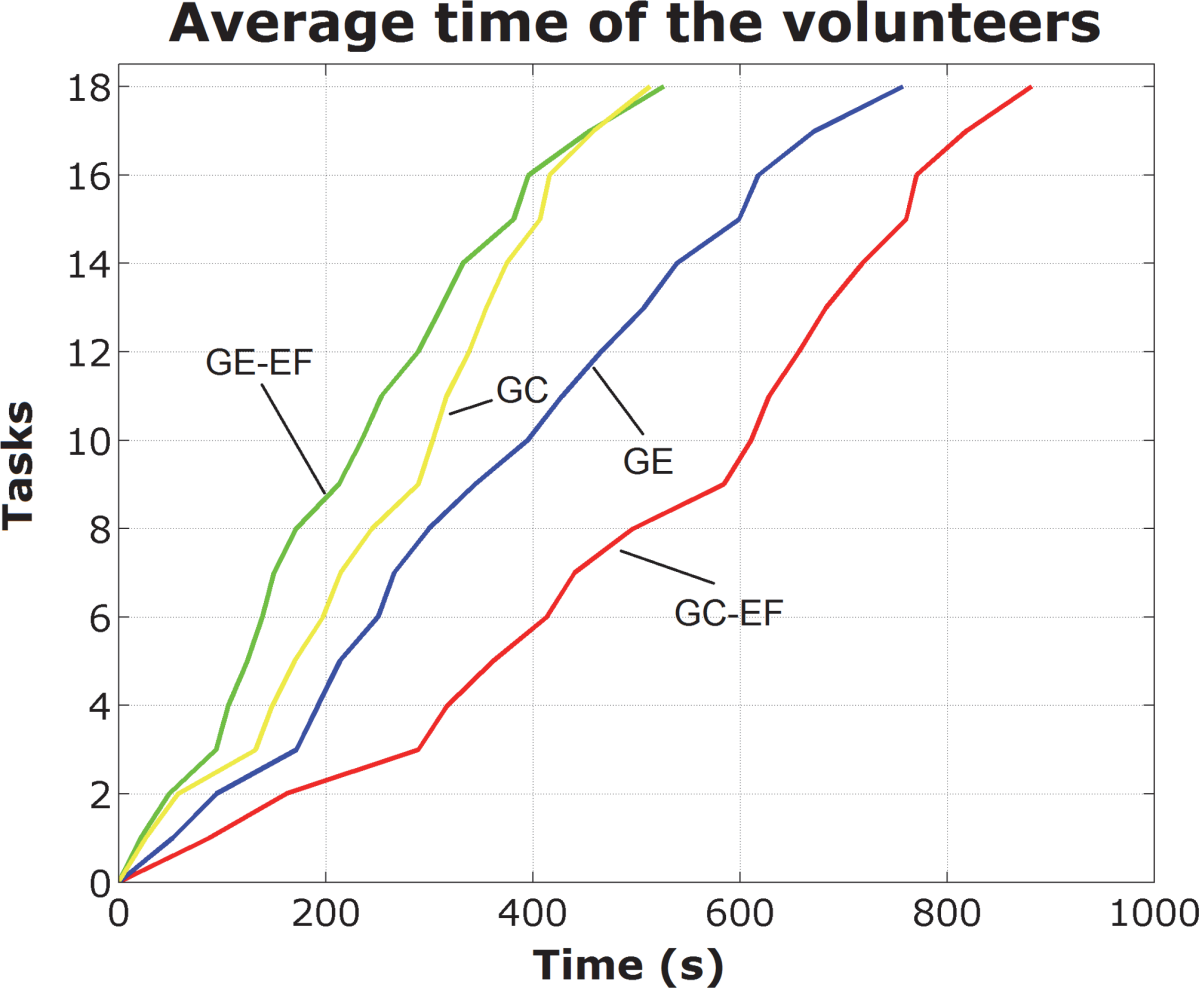
Graph of the average performance of the 4 subgroups.

As the time for performing each task was obtained discretely, the Matlab software was used to construct computational routines that allow generating continuity depending on time (Fig 7).

## Discussion

The results of this study showed that the GE-EF and GC groups overall had similar performances. The software's second stage (tasks 9 to 18) was contextualized in a minefield, where the player with better concentration throughout the tasks is favored (in this case GC had high-performance). Therefore, the volunteers from the GE-EF presented better results in the software's first stage (tasks 1–9), which suggests that this phase is favored by an exploratory behavior, which is an intrinsic characteristic of the subjects with ADHD.

Many variables can affect the collected information in computer games, such as ability to use the command interface, intensive practice in computer games or even lack of collaboration and intellectual level of participants [4]. In our study, we selected volunteers who had similar skills in games and academic performance. The students with the best and worst grades in several disciplines were left out of this study. This step allowed for a standard derivation of less than 5% for task execution time in the same group. Therefore, we believe that our method is not susceptible to uncontrolled variables.

The results of this study also show that the GC group has a performance that is 42% better than the GC-EF group. These data seem indicate that after an intense physical exercise, people without ADHD show impairment in attention performance. However, this hypothesis requires further studies, which include the assessment of the duration of this effect.

The computer game “Raiders of the Lost Treasure” [11] allowed us to generate measurable records and showed the improved attention of the students with ADHD that experienced intense exercise in a short time interval (approximately 5 min). Therefore, children with ADHD symptoms may have equivalent concentration to individuals without the disorder after physical exertion. Over past decades, several studies on humans [9, 17, 18] have demonstrated the benefits of exercise on health and brain function. These authors clarify that regular exercise can protect the brain against disease and certain types of brain injuries because many positive effects occur in the hippocampus, which is important region for learning and memory.

Lim et al. [19] used a training system with a more intensive game for 20 unmedicated children with ADHD and significant symptoms of inattention. The treatment consisted of eight weeks of training with 24 sessions followed by three monthly booster training sessions. In Lim et al. [20], the game for attention training was considered a new potential treatment for ADHD.

Fuermaier et al. [12] explored the effects of whole-body vibration on attention in healthy individuals and adults who were diagnosed with ADHD. The authors used the beneficial effects of whole-body vibration on various physiological measures. Treatment was passively performed while the volunteers were seated in a chair that was placed on a vibration platform. Fuermaier et al. [12] showed significant beneficial effects of small to medium size for all volunteers and improved cognitive performance of healthy volunteers and those with ADHD after 2 min of treatment. All volunteers in this study were subjected to intense physical exercise, which increased their maximum heart rate from 65% to 85% and made them tired and euphoric in the end. Physical activity increases the release of serotonin, dopamine and norepinephrine, and the increase in these neurotransmitters makes the normal volunteers more excited after the physical activity. With their decrease, the volunteers calm down and return to focus and have normalized attention. Therefore, it is assumed that the attention of the subjects with ADHD is improved because of the release of these neurotransmitters. These data corroborate with other studies [21], which report that physical exercise helps building the mechanism that increases the amount of neurotransmitters in the brain and their postsynaptic receptors. During the physical activity, more neurotransmitters (dopamine, serotonin and norepinephrine, as well as other molecules) are released and may regulate this imbalance. Exercise improves the mood, well-being, anxiety and depression and helps one cope better with stress.

A physical education survey [18] observed that physical exercise can reduce anxiety symptoms. The effect was independent of physical fitness, although the anxiety levels were lower in the group that achieved better aerobic fitness during the treatment.

Medina et al. [9] examined the possibility that physical exercise improved the sustained attention in children with ADHD and tested the hypothesis of the interaction of catecholamines and their receptors in the prefrontal cortex and striatal areas in cognitive improvement that was acutely induced by effort. The children's attention immediately after exercise was compared to assess the chronic effects of psychostimulants and whether they caused some change in test-II Conner's Continuous Performance (CPT) immediately after exercise. The results of Medina et al. [9] showed that exercise resulted in higher alertness, decreased impulsiveness, increased reaction speed and greater stability in all aforementioned metrics. Our study shows that physical activity also improves the performance of children in tasks that require attention.

## Conclusion

This study confirms the hypothesis of Medina et al. [9] and Koehl et al. [10]: intense physical exercise improves the attention of children and adolescents with ADHD symptoms. Physical exercise may be helpful for their learning because attention is essential to the school performance of any individual.

Physical exercises help improve children's attention and provide greater impulse control; these additional effects appear almost immediately, as confirmed in this study, which helps the concentration of children with ADHD.
